# Cell biology of the chick organizer: Origins, composition, population dynamics and fate^☆^

**DOI:** 10.1016/j.cdev.2025.204017

**Published:** 2025-03-06

**Authors:** Claudio D. Stern

**Affiliations:** Department of Cell & Developmental Biology, University College London, Gower Street, London WC1E 6BT, UK

**Keywords:** Chicken, Chicken embryo, Hensen’s node, Gastrulation, Neural induction, Patterning

## Abstract

The year 2024 celebrates 100 years of perhaps one of the most important and influential papers in the field of developmental biology: Spemann and Mangold’s publication reporting the discovery of the “organizer”, which can induce and pattern the nervous system and also pattern the axial-lateral axis of the mesoderm. While many papers have investigated, and many others reviewed, the signalling aspects of the organizer, relatively fewer have concentrated on the cell biology of organizer cells. Here we survey more than 12 decades of knowledge on the chick organizer, including the cellular origins, fates, composition, cell movements, cell population properties and molecular dynamics of the chick organizer (the tip of the primitive streak). What emerges is a picture of an extremely complex and dynamic population of cells whose properties change over space and time, quite different from the “textbook” view of a static group of cells set aside during early development to perform a particular function in the normal embryo before being swept aside. Some of these findings also have more general implications for the interpretation of results from single cell RNA sequencing experiments.

## Introduction

Not long after the historic 1924 paper by Hans Spemann and Hilde Mangold reporting the discovery of the organizer (Spemann and Mangold, 1924) that is being celebrated by this special issue, experiments by C.H. Waddington identified the tip of the primitive streak of the chick and duck embryo as a functionally equivalent region (Waddington, 1930; Waddington, 1932; Waddington, 1933; Waddington, 1934; Waddington and Schmidt, 1933). When transplanted into an ectopic region of a host embryo, the graft would induce the formation of an ectopic, fully patterned neural plate from host ectoderm cells. These and many other papers that sprouted up over the next 2–3 decades helped to define key concepts that set the foundations of classical Developmental Biology as a discipline. Many of these concepts were particularly clearly articulated by Waddington (Stern, 2004; Waddington, 1966; Slack, 1983), whose sharply quantitative mind helped him to arrive at definitions with quasi-mathematical precision and clarity (Stern, 2000). An important distinction is that between the activity of a tissue that has inductive properties, which emits signals that can change the direction of differentiation (i.e. the fate, or cell type identity) of a responding tissue (Gurdon, 1987), and a tissue that has the full attributes of a true “organizer”. The latter can not only *induce* the responding tissue, but must also be able to *pattern* its derivatives into a spatially coherent set of structures (Anderson and Stern, 2016). This definition is entirely reliant on the properties of the signalling and responding tissues after an experimental manipulation that brings them into juxtaposition under clearly defined conditions, and does not necessarily reflect what these tissues do in a normal, undisturbed embryo. In fact, many of the concepts of developmental biology require clear distinction between what a group of cells normally *does* during development (for example their fate), and what the same group of cells *can do* (their *developmental potential*, now often called *potency*) – it is the difference between these two sets of properties that allows the design of more elegant experiments characteristic of *Entwicklungsmechanik* (“experimental embryology”) (Stern, 2004; Slack, 1983).

The properties of the archetypal, “Spemann-Mangold” organizer have been very extensively reviewed over these 10 decades, especially relating to its signalling functions (Hamburger, 1988; Nakamura and Toivonen, 1978; Stern, 2024; Harland and Gerhart, 1997; De Robertis and Kuroda, 2004; Gallera, 1971). Three main properties characterise the Spemann organizer across the vertebrates: its ability to induce a neural plate from ectoderm cells not fated to contribute to this tissue, its ability to pattern the resulting neural plate along its head-tail and dorsoventral axes, and its ability to pattern the non-axial mesoderm into “ventral” (lateral) and more “dorsal” (medial) derivatives. The most axial mesoderm (chordamesoderm) is derived from the organizer itself, whereas the rest of the mesoderm (extraembryonic, lateral plate, intermediate mesoderm, heart, and paraxial presomitic and head mesoderm) is subdivided by signals emanating from the organizer and its derivatives.

As reviewed by other contributions to this volume, the organizer resides in the dorsal lip of the blastopore of anuran and urodele amphibians, the embryonic shield of teleost fishes, and the tip of the primitive streak in bird and mammalian embryos. In the latter vertebrate Classes, the organizer and some of its derivatives have an additional signalling function: setting up the initial cues for left-right asymmetry. The present review concentrates on the avian organizer. As the signalling properties of this have recently been discussed extensively elsewhere (Stern, 2024), the present review concentrates on the cellular aspects of the organizer in the chick, an area that has not yet received enough attention.

**Fig. 1.**
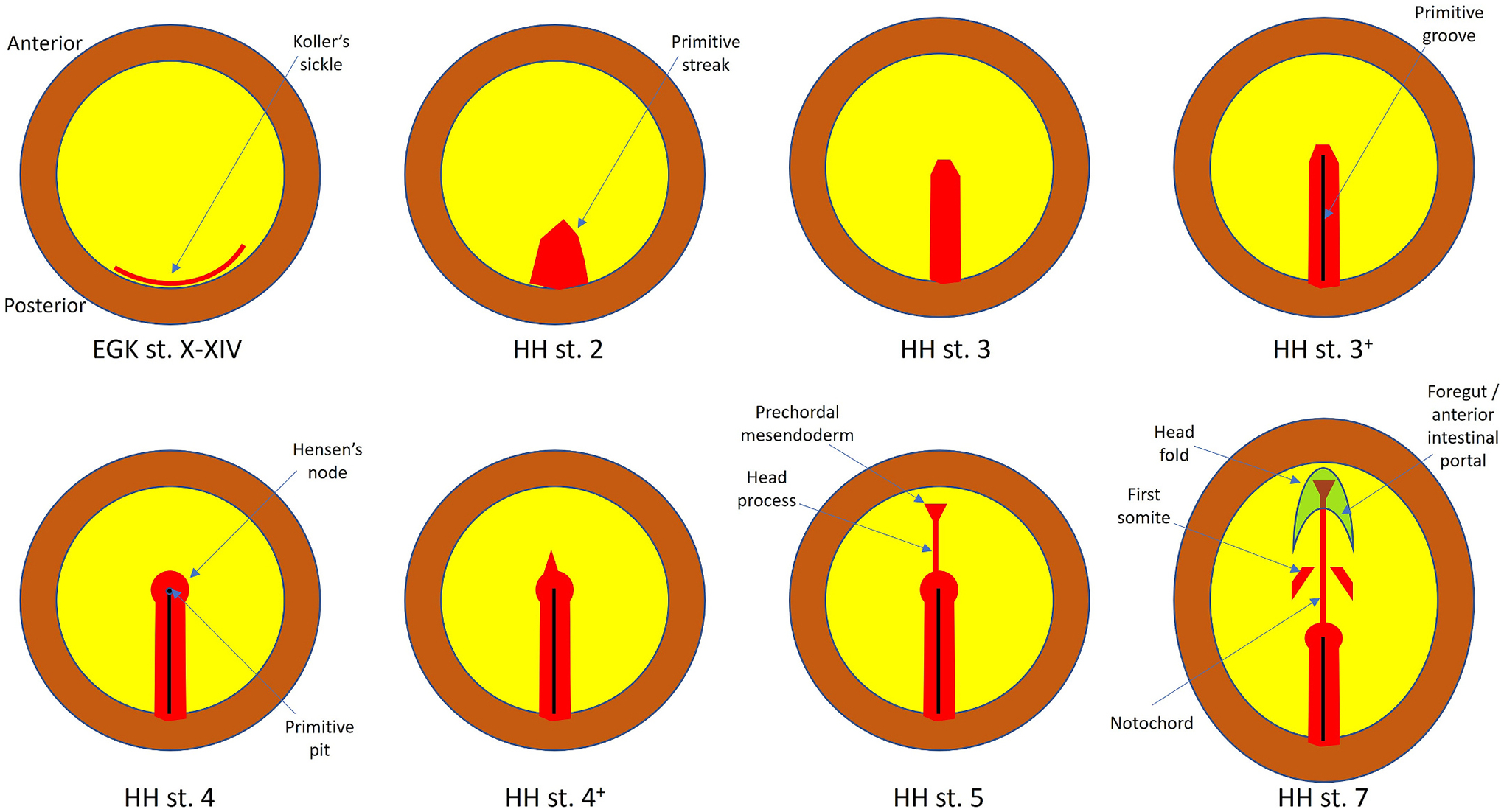
Stages of early chick development referred to in the text. The diagrams show some of the features used to classify the stages in relation to primitive streak formation. EGK, stage according to Eyal-Giladi and Kochav (Eyal-Giladi and Kochav, 1976) (in Roman numerals; applies to EGK stages I-IX which take place within the oviduct, stage X, early blastoderm, approximately when the egg is laid, to EGK stage XIV just before the primitive streak starts to appear). Here a single diagram represents EGK stages X-XIV and details are not shown. HH, stage according to Hamburger and Hamilton (Hamburger and Hamilton, 1951) (applies from HH stage 2, when the primitive streak first appears, to hatching 20 or so days later). Brown: extraembryonic area opaca at the periphery of the blastoderm. Yellow: epiblast of the area pellucida in the centre of the blastoderm. Red: mesoderm/mesendoderm. Red crescent at early stages: Koller’s sickle. Red rod in the midline: primitive streak. Hensen’s node (red circle at the tip of the primitive streak) appears at HH4. At stage HH4+ a triangular shape appears corresponding to the future prechordal mesendoderm. At stage HH5 the head process emerges from the node, with the prechordal mesendoderm at its tip. After this the primitive streak starts to regress and the notochord elongates caudally in its wake as the node becomes incorporated with the shrinking primitive streak into the forming tail bud. “Organizer” functions are localised at the tip of the primitive streak from stage HH 3+ (grooved primitive streak) to stage HH 4 (appearance of Hensen’s node but before emergence of prechordal cells).

The tip of the primitive streak of the chick embryo has the ability to induce a fully patterned nervous system from competent cells only at the fully elongated primitive streak stage, from Hamburger and Hamilton (HH) stage 3 (Hamburger and Hamilton, 1951) to just before a morphological node emerges at late HH stage 4 (Fig. 1). As soon as prospective prechordal mesendoderm cells emerge from the tip (HH stage 4^+^), its ability to induce starts to decrease, and the induced nervous system lacks the most anterior parts (forebrain) (Gallera, 1971; Dias and Schoenwolf, 1990; Storey et al., 1992). Until stage HH4^−^, the tip of the streak is continuous with the body of the streak and there is no obvious morphological boundary that defines it caudally, other than relative to the position of the primitive pit. At stage HH4, the tip of the primitive streak acquires a bulbous shape, similar to what was originally described as a “Knoten” (knot, or node) in guinea pig and rabbit embryos by Viktor Hensen (Hensen, 1876). For this reason, this bulbous structure is commonly referred to as “Hensen’s node”. However, the full “organizer” properties of the primitive streak start to be lost just as the node becomes visible as such.

## Molecular markers for the organizer

The first molecular marker for the organizer to be identified in any vertebrate was the homeobox gene *Goosecoid (*Cho et al., *1991*; Blumberg et al., *1991**)*, in Xenopus. Soon, orthologs were discovered in mouse (Blum et al., 1992), chick (GSC) (Izpisúa-Belmonte et al., 1993), zebrafish (Stachel et al., 1993) and human (Blum et al., 1994), and throughout the vertebrates. Other transcription factors expressed in the same region followed, including *FOXA2* (originally *HNF3β*) (McMahon, 1994; Collignon et al., 1996; Ruiz i Altaba and Jessell, 1992), *NOT* (von Dassow et al., 1993; Talbot et al., 1995; Knezevic et al., 1995) and many others (De Robertis et al., 1994). The organizer also expresses genes encoding many other types of proteins, of which signalling molecules have attracted particular interest because of its obvious non-cell-autonomous functions (Anderson and Stern, 2016; Sasai et al., 1996; Sasai et al., 1994; Anderson et al., 2016; Streit et al., 1998) (see previous sections). There are differences in the temporal patterns of expression of these genes with development. Some of them, notably *GSC*, are expressed only up to the stage when the organizer has full inducing properties (stage HH4 in the chick) (Hamburger and Hamilton, 1951; Dias and Schoenwolf, 1990; Storey et al., 1992) and is then quickly downregulated (Izpisúa-Belmonte et al., 1993). Others, such as *FOXA2*, *NOT* and *Chordin (CHRD)* are expressed in the organizer as well as in its axial derivatives, the chordamesoderm/notochord and remains expressed in the late node and tail bud (see (Anderson and Stern, 2016; Anderson et al., 2016; Streit et al., 1998)). Many genes that are expressed in the organizer are also expressed in other structures that are not progenitors or descendants of organizer cells at other stages of development. Among them, the transcription factors *NOT1* and *NOT2* (Knezevic et al., 1995; Knezevic and Mackem, 2001) and *OTX2* (Foley et al., 2000; Bally-Cuif et al., 1995; Albazerchi and Stern, 2007) share an initial broad expression pattern in the epiblast prior to primitive streak formation (regulated by the underlying hypoblast, (Knezevic and Mackem, 2001; Foley et al., 2000)), then become expressed strongly at the tip of the primitive streak and node, then diverge, with *NOT1* and *NOT2* remaining in Hensen’s node and the notochord that emerges from the node, whereas *OTX2* is downregulated from all node derivatives and becomes expressed in the anterior epiblast, and later in the forebrain and midbrain regions of the neural tube (Foley et al., 2000; Bally-Cuif et al., 1995; Simeone et al., 1992). Even *GSC* itself, the original archetypal molecular label for the organizer, is expressed earlier in development (in Koller’s sickle and in the hypoblast), then in the prechordal mesendoderm at neurulation stages and later in other unrelated structures (Izpisúa-Belmonte et al., 1993; Zhu et al., 1999a). These examples clearly illustrate that there is no single gene that represents a true, un-ambiguous and exclusive “marker” of the organizer, and also that there is no single gene that marks the progenitors of the organizer, the organizer itself, and cells derived from it – this will be discussed further below.

A related question is whether any particular subset of genes is co-expressed in the organizer, and/or in other signalling centres that have organizer properties at other times and places during development. As techniques for high throughput mRNA analysis became more widely available and affordable, this question became more tractable. In one study, the transcriptomes of three tissues with inducing and patterning (i.e. organizer) functions (the tip of the primitive streak, the notochord/floor plate and the zone of polarizing activity of the limb bud) were compared with each other and also with the most similar tissue that lacks those functions (Anderson et al., 2016). The analysis identified a small subset of genes that are co-expressed in all three of these inducing/patterning regions; the expression of these genes was then studied over a broad range of stages, to seek for other regions where a significant number of them are also co-expressed but which had not been identified as organizers. This strategy revealed at least one candidate region, the anterior intestinal portal endoderm (AIP), and it was then shown that it has inducing and patterning properties on the adjacent prospective heart mesoderm (Anderson et al., 2016). Although the study also hinted at other regions that could have signalling and patterning properties, it seems likely that these are rare cases and that other organizers, if they exist, may not share enough of their molecular signature for this to be a viable strategy (Anderson and Stern, 2016; Anderson et al., 2016).

## Origin of the cells contributing to the organizer

**Fig. 2.**
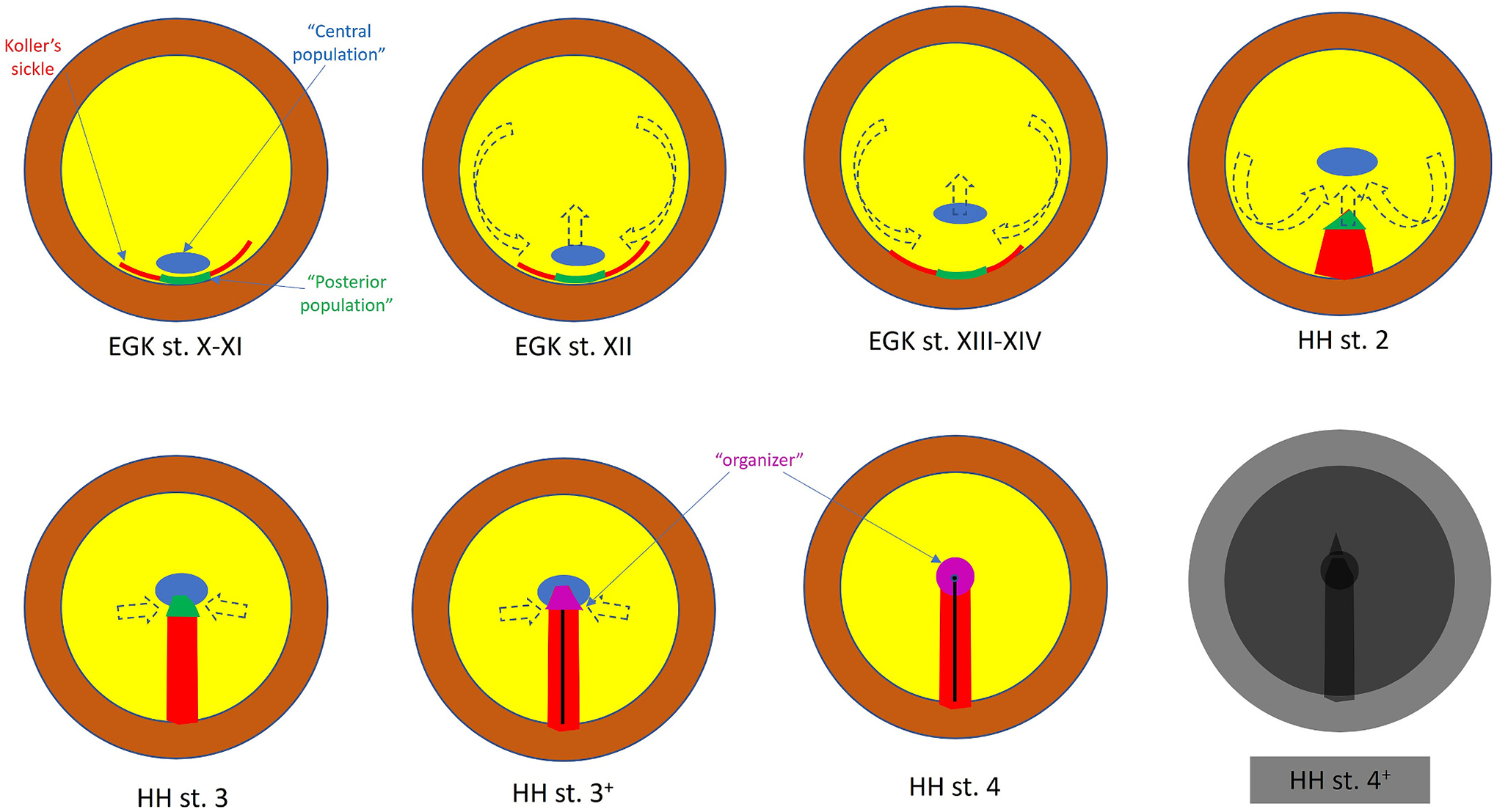
Cell movements of populations contributing to the organizer. The diagrams show embryos at successive stages from EGK X-XIV and HH 2–4, showing the origin and movements of cells that make up the organizer at different stages. Colour key as for Fig. 1. Green: “posterior” organizer precursor cells arising from the middle of Koller’s sickle. Blue: “central” population of organizer precursors, arising from epiblast just adjacent to the sickle at stage X, which then move to the middle of the embryo prior to primitive streak formation. As the streak forms, green Koller’s sickle derived cells catch up with the central population and organizer properties (purple) arise. These organizer properties last until stage 4^+^, when prechordal mesendoderm cells start to leave the node. The embryo after this stage is showed “greyed out” to symbolise the loss of these properties. Thick arrows show the predominant cell movements before gastrulation (“Polonaise”, EGK stages XII-XIV), primitive streak formation and elongation (HH stages 2–3^+^) and subsequent regression (HH stages 5 onwards).

When examining the changing expression of *GSC* throughout early development, we originally noticed that at pre-primitive-streak stages, it is expressed in Koller’s sickle, a ridge of cells projecting ventrally from the posterior epiblast, which demarcates the boundary between the area pellucida centrally and the marginal zone and area opaca peripherally (Izpisúa-Belmonte et al., 1993) (Fig. 2). As the primitive streak forms and elongates towards the centre of the embryo, the expression tracks the cells at the tip of the extending primitive streak, becoming localised to the region corresponding to the organizer based on functional (grafting) criteria, until stage HH4, the full primitive streak stage. This raised the question of whether the initial *GSC* expressing cells are precursors, and if so the only precursors, of the organizer. To investigate this, we first marked a subset of *GSC* expressing cells in the sickle using the carbocyanine dye DiI, and incubated embryos to stage HH4; all DiI-labelled cells ended up within the tip of the primitive streak and within the population expressing *GSC* at this later stage, suggesting that sickle cells indeed contribute to the organizer (Izpisúa-Belmonte et al., 1993). However, many *GSC*-expressing cells did not have DiI fluorescence. This is either because only a subset of sickle cells had been marked, or because not all cells that make up the organizer are derived from the *GSC*-positive population in the sickle (the “posterior population” – Fig. 2). A first experiment to answer this question was designed to test whether *GSC*-expressing cells could recruit non-expressing neighbours and induce them to express. Expressing cells from a quail donor embryo were grafted to an ectopic site in a chick host, and embryos incubated until the primitive streak had formed. Many embryos formed an ectopic streak, containing both some quail cells and some chick cells that were now expressing *GSC*, suggesting that indeed, the organizer recruits cells into itself which then become part of the organizer (Izpisúa-Belmonte et al., 1993). Fate maps of the early embryo (Hatada and Stern, 1994; Rosenquist, 1966; Rosenquist, 1971; Rosenquist, 1983; Psychoyos and Stern, 1996a; Bachvarova et al., 1998; Schoenwolf and Sheard, 1990), together with analysis of the patterns of movements of epiblast cells during primitive streak formation (Voiculescu et al., 2007; Voiculescu et al., 2014; Gräper, 1929; Wetzel, 1929) showed that the tip of the primitive streak recruits cells from the neighbouring epiblast as it elongates, and that this process ceases just after stage HH4, when a morphological Hensen’s node appears as a bulbous thickening of the tip (Sheng et al., 2003; Solovieva et al., 2022a).

More targeted experiments to identify the region of origin of organizer cells that are not derived from the sickle revealed that a population of epiblast cells which is initially (at pre-primitive-streak stage EGK X, (Eyal-Giladi and Kochav, 1976)) adjacent to the sickle moves anteriorly within the epiblast, along the midline, ending up around the site at which the tip of the streak will come to rest at stage HH4 (Foley et al., 2000; Streit et al., 2000). This anterior movement is part of the “Polonaise” movements of the epiblast that begins prior to primitive streak formation (at stage EGK XI-XII) (Voiculescu et al., 2007; Voiculescu et al., 2014; Gräper, 1929; Wetzel, 1929), causing this “central population” of node precursors (Fig. 2) to move ahead of the tip of the primitive streak (Foley et al., 2000; Streit et al., 2000). Grafting either the sickle (“posterior population”) or the “central population” of organizer precursors alone in a neural induction assay shows that neither alone induces neural plate markers, but the combination of the two does, suggesting that the dual cellular origin of the organizer also involves the juxtaposition of two (or more) different cellular functions required for neural induction (Streit et al., 2000).

## Fate of cells in the organizer

**Fig. 3.**
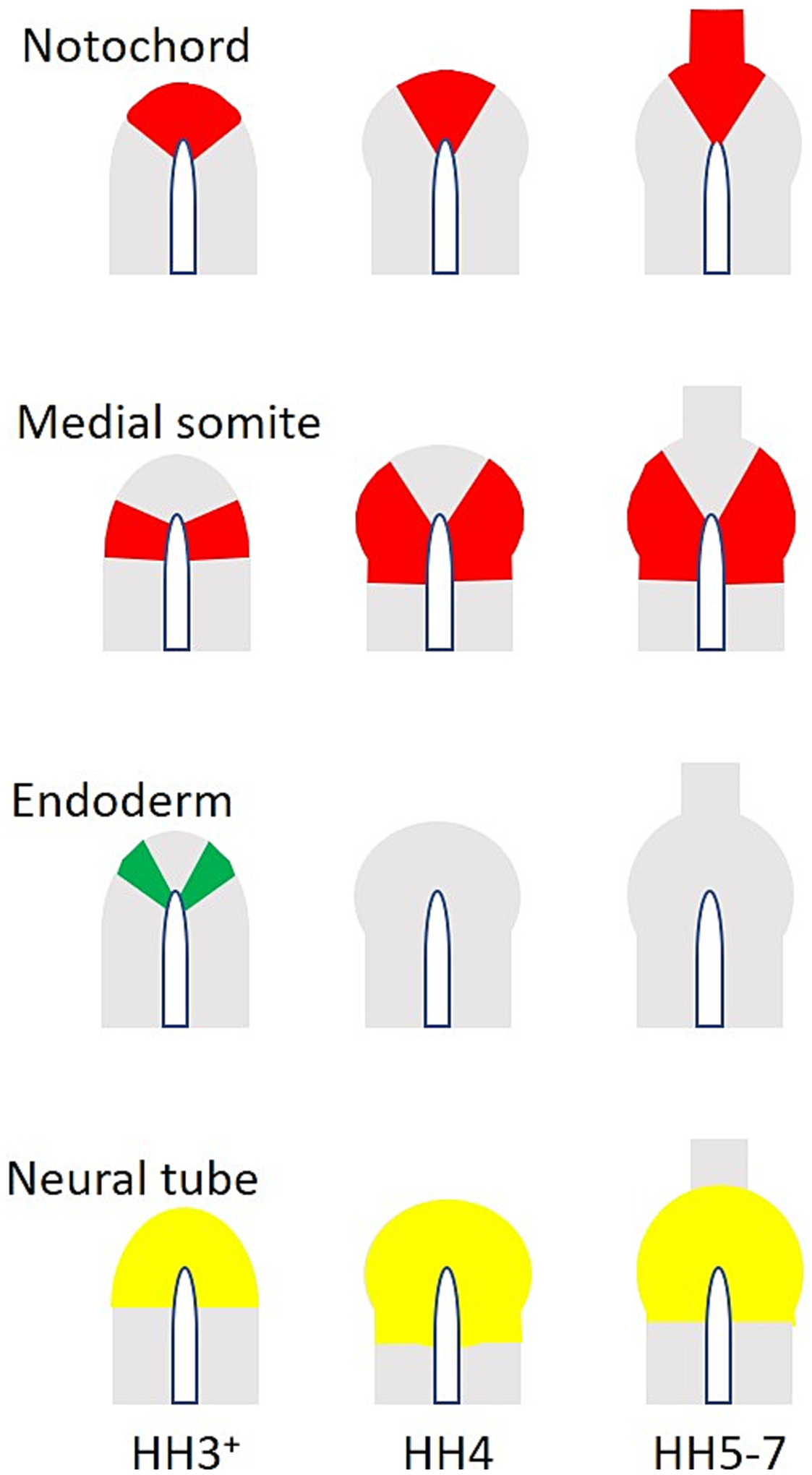
Fate maps of the ectodermal (dorsal) layer of the organizer/node, based on Selleck and Stern (1991) (Selleck and Stern, 1991). Fate maps are drawn for three stages: HH 3^+^, HH 4, and HH 5–7 (columns). Each row summarises the distribution of precursors for the main tissues arising from the epiblast part of the organizer/node: notochord, medial somite, endoderm and neural tube/floor plate. Mesodermal tissues are shown in red, endoderm in green and ectodermal derivatives in yellow.

Numerous fate mapping studies, based on transplantation to construct quail-chick chimaeras, or from radiolabelled donor embryos, or carbocyanine-dye labelled donors, or by local labelling with dyes or other methods, have been conducted over many decades, leading to a good understanding of the destination of cells arising from the tip of the primitive streak at various stages (summarised in Fig. 3). At stage HH4^−^, shortly before it acquires a bulbous morphology to become the typical “Hensen’s node”, the tip of the streak contains precursors for the prechordal mesendoderm, notochord (including head process and trunk portion of the chordamesoderm), the medial part of the floor plate of the neural tube, definitive (gut) endoderm and somites (Rosenquist, 1966; Rosenquist, 1971; Rosenquist, 1983; Psychoyos and Stern, 1996a; Schoenwolf and Sheard, 1990; Wetzel, 1929; Sheng et al., 2003; Solovieva et al., 2022a; Bellairs, 1986; Lawson and Schoenwolf, 2001; Lawson and Schoenwolf, 2003; Lopez-Sanchez et al., 2001; Rosenquist, 1970; Schoenwolf et al., 1989; Schoenwolf et al., 1992; Schoenwolf and Yuan, 1995; Selleck and Stern, 1991; Bellairs, 1953a; Bellairs, 1953b; Spratt, 1946; Spratt, 1947; Spratt and Condon, 1947; Kimura et al., 2006; Kirby et al., 2003). After stage HH4, the node no longer contains precursors of the endoderm and recruitment of cells from the neighbouring epiblast ceases (Sheng et al., 2003; Solovieva et al., 2022a; Schoenwolf et al., 1992; Kimura et al., 2006). The first axial cells to emerge, in an anterior direction from the node, are the precursors of the prechordal mesendoderm, at stage HH4^+^ (Izpisúa-Belmonte et al., 1993; Rosenquist, 1966; Rosenquist, 1983; Psychoyos and Stern, 1996a; Sheng et al., 2003; Schoenwolf et al., 1992; Selleck and Stern, 1991; Spratt, 1947; Spratt and Condon, 1947; Schoenwolf, 1992) (Fig. 3).

**Fig. 4.**
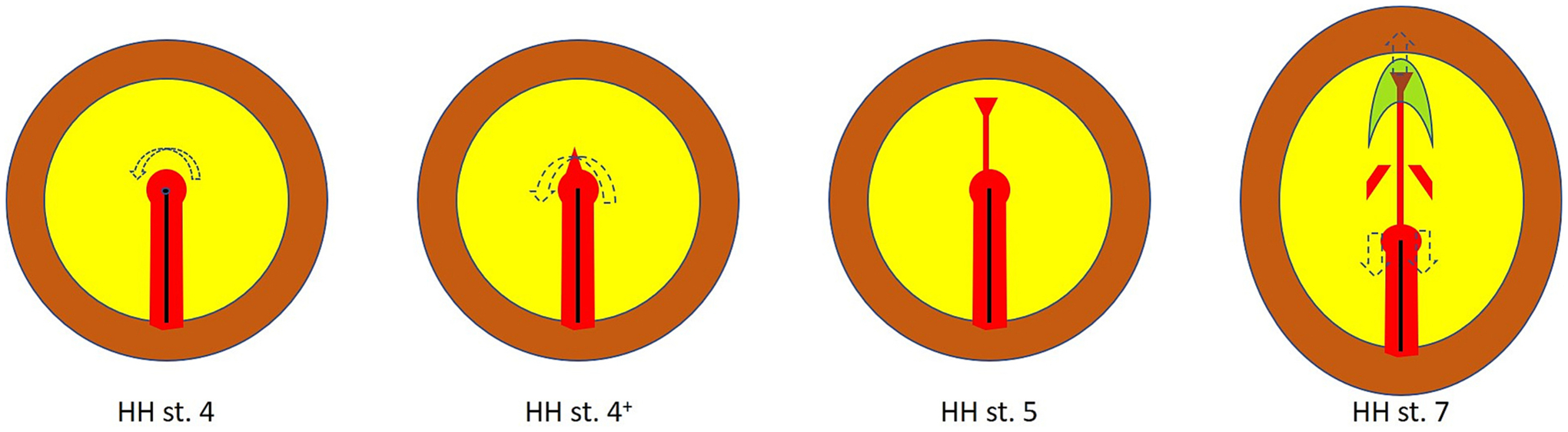
Cell movements around the organizer/node after stage HH4. The arrows show the predominant cell movements (viewed from the epiblast side). A leftward rotation results in asymmetric positioning of the site of formation of prechordal and chordamesoderm cells towards the left side of the embryo. After stage 5, the node regresses back into the primitive streak to make up the tail bud as the cranial end of the embryo moves in the opposite direction, driven by convergence extension movements at the midline of the epiblast and chordamesoderm which elongate the embryo while also narrowing it mediolaterally.

As the node regresses down the primitive streak, these two structures together become established as the “tail bud” (Solovieva et al., 2022a; Spratt, 1947; Spratt and Condon, 1947; Iimura et al., 2007; McGrew et al., 2008; Solovieva et al., 2022b), which continues to contribute to the trunk notochord and somite mesoderm along the remaining length of the embryonic axis (Fig. 3–4). The contribution to the medial floor plate appears to cease around the time at which neurulation starts (stage HH5–6), when the node is located in the prospective posterior hindbrain (around rhombomeres 5–6, the level of the otic vesicle); the remainder of the floor plate (caudal hindbrain and trunk) is largely the result of induction of the overlying ectoderm by the notochord (Schoenwolf and Sheard, 1990; Lopez-Sanchez et al., 2001; Schoenwolf et al., 1989; Schoenwolf et al., 1992; Schoenwolf and Yuan, 1995; Schoenwolf, 1992; Dodd et al., 1998; Placzek et al., 2000; van Straaten and Hekking, 1991; van Straaten et al., 1985; van Straaten et al., 1988). When a node is isolated and cultured in the coelomic cavity of a host embryo, it still gives rise to its normal axial derivatives (Leikola, 1978).

Although the tip of the primitive streak and the node contain precursors of the pre-somitic mesoderm (PSM) and the resulting somites, not all of the paraxial mesoderm is derived from it. Specifically, the lateral sectors of the node contain precursors of only the medial halves of the somites (Selleck and Stern, 1991), which in turn will contribute to epaxial (dorsal) skeletal muscles of the trunk (Ordahl and Le Douarin, 1992), whereas the lateral half is derived from cells ingressing through the primitive streak just posterior (caudal) to the node (Psychoyos and Stern, 1996a) – this half will contribute to the hypaxial (ventral) skeletal musculature. A sharp line seems to demarcate the boundary between these two compartments within the somite. Both halves largely contribute to other somite derivatives including the intervertebral discs and vertebral bodies and other structures, although this is subsequently complicated further a little by a 45° rotation of the somites at later stages. However sharp, the different origins of the medial and lateral halves of the somites are not the only cause of the differences between these somite populations – signalling from neighbouring tissues, most significantly from BMP4 (from the lateral mesoderm), BMP inhibitors and Shh (both emanating from the notochord and floor plate) and perhaps Wnt from the dorsal ectoderm is particularly important (Pourquie et al., 1996; Streit and Stern, 1999). In fact, the strength of BMP signals is sufficient, even after formation of the PSM, to convert the entire PSM into lateral mesoderm (when BMP is high), and vice-versa (Streit and Stern, 1999; Dias et al., 2014; Tonegawa et al., 1997; Tonegawa and Takahashi, 1998), indicating that the node or primitive streak origin of these tissues is not as important as the signals they receive in determining their ultimate identity. Indeed, the organizer itself, as a source of BMP antagonists, has strong dorsalising activity on the mesoderm, which was one of the properties that defined the organizer in amphibians (Harland and Gerhart, 1997; De Robertis and Kuroda, 2004; Bier and De Robertis, 2015). In the chick, transplantation of a node into the lateral plate mesoderm, or co-grafts of the node together with precursors of lateral mesoderm, can generate somites from the lateral mesoderm (non-somite-fated) tissue (Dias et al., 2014; Tonegawa et al., 1997; Tonegawa and Takahashi, 1998; Nicolet, 1968; Nicolet, 1970; Nicolet, 1971; Hornbruch et al., 1979). Interestingly, not all BMP inhibitors expressed in the node have the same ability to convert lateral mesoderm into somites: it seems to be a property particularly associated with Noggin, which starts to be expressed relatively late (HH4/4^+^) (Streit and Stern, 1999). Overall, the environment in which node derivatives find themselves after leaving the organizer is important for determining their identity, especially for paraxial mesoderm, but the notochord seems particularly resilient to external signals (Streit et al., 1998; Leikola, 1978; Streit and Stern, 1999).

## Cellular composition of the organizer: Resident and transitory cell populations

After the full primitive streak stage (HH stage 4), the first axial mesendoderm cells emerge by migrating anteriorly from the medial tip of the node. They are the precursors of the prechordal mesendoderm and the “head process”, a name given to the chordamesoderm/notochord underlying the future brain, down to the mid-hindbrain level (Rosenquist, 1966; Rosenquist, 1983; Schoenwolf et al., 1992; Spratt, 1947; Spratt and Condon, 1947; Seifert et al., 1993; Sausedo and Schoenwolf, 1993). This defines stage HH5 (Fig. 1). Immediately thereafter, as the head fold forms (defining the foregut and anterior intestinal portal endoderm) (stage HH6), regression of the node in a caudal direction begins. Classical studies using labelling with carbon particles established that this caudal movement is largely the result of real caudal migration of its cells, although the “node property” also regresses, incorporating some cells from the primitive streak as the node moves into the latter to make up the shortening tail bud (Spratt, 1947; Spratt and Condon, 1947). Spratt suggested that the main driving forces for this caudally-directed movement are mainly located in more anterior regions of the blastoderm. The idea that the node contains resident cells was also supported by grafting labelled whole nodes into an unlabelled host – as the node generates labelled descendants, the node itself remains labelled until late stages when it is located in the tail bud (Solovieva et al., 2022a; McGrew et al., 2008; Solovieva et al., 2022b; Wood et al., 2024). Recruitment of cells into the node from the neighbouring (lateral, or “paranodal”) epiblast ceases at HH4/4^+^ (Sheng et al., 2003; Spratt, 1947; Spratt and Condon, 1947).

Evidence for the idea that the anterior primitive streak and node contain a “resident” population of cells was strengthened by following the lineage descendants of single node cells marked by intracellular injection of lysine-rhodamine-dextran (Selleck and Stern, 1991). Different sub-regions of the node were found to contribute to distinct derivatives: the median anterior portion giving rise to chordamesoderm, the lateral/posterior portions to paraxial (including pre-somite) mesoderm, and the region around the tip of the groove up to stage HH4^−^ giving rise to the definitive gut endoderm. Strikingly, in some cases, an individual labelled cell in the epiblast was found to generate clusters of descendants along the axis of the notochord (about 2–3 somite-lengths apart) or in the pre-somitic mesoderm and somites (about 6–7 somites apart). In one occasion, a single cell generated both of these types of clusters. When clusters were observed, the number of cells making up each cluster decreased caudally in an approximately exponential manner, with just one labelled cell remaining at the node itself (Selleck and Stern, 1991; Selleck and Stern, 1992). The most parsimonious interpretation of these findings is that the epiblast of the primitive streak/node contains a resident population of cells that follow the node during its regression, and that at each cell division, one daughter leaves the node (acting as a founder of a cluster) while its sister remains in the node and repeats the process at each cell division. This is typical “stem cell” behaviour, with asymmetric divisions generating one self-renewing daughter (remaining in the node) and a differentiating sister (Selleck and Stern, 1992; Stern et al., 1988).

Serial homotopic transplantation of the node/tail bud across a series of successively younger host embryos demonstrates the self-renewing nature of the node cell population (McGrew et al., 2008). This has also recently been demonstrated at the level of a single cell (Solovieva et al., 2022a). Importantly, the latter study showed that the property of being a resident stem-cell-like cell is not associated with a specific preexisting population of node cells, but that at least some cells that are incorporated into the node can acquire this property upon entering this structure, and that even epiblast cells from a remote area (which would never normally have contributed to the node) can be endowed with this property if placed within the paranodal territory that will be absorbed into the node (Solovieva et al., 2022a). This suggested that the node is, or contains, an informative “niche” that can capture cells and retain them thereafter through successive asymmetric divisions (Solovieva et al., 2022a; Solovieva et al., 2022b) – time-lapse filming of a mosaic of cells labelled by electroporation of fluorescent proteins suggested that the niche is located in the posterior-lateral sectors of the node (Solovieva et al., 2022a).

Single cell RNA sequencing of epiblast derived cells “captured” by the node that then became resident revealed that some genes associated with the cell cycle, and more specifically the G2/M transition, are enriched in those cells in comparison with their non-resident neighbours (Solovieva et al., 2022a). Since dividing cells are present throughout the node, this concentration of cells at the G2/M transition in the posterior-lateral part of the epiblast raises the possibility that there are extensive cell movements even within this epithelial sheet, perhaps placing cells that are about to divide close to the posterior-lateral boundary of the node to allow asymmetric cell division to occur, shedding only one of the daughters of the division. However, this behaviour has not yet been observed directly. More detailed time-lapse analysis of cell behaviours throughout the node, especially using techniques that allow cells to be followed throughout the entire thickness of this compact structure, will be required to resolve this.

## The organizer as a location in the embryo rather than a cell population: Gene expression marks cell states, not cell fates

Ablation of the organizer region at the primitive streak stage leads to complete reconstitution of the organizer within 6–9 h, including normal gene expression, morphology and formation of axial and paraxial derivatives (Psychoyos and Stern, 1996b; Yuan et al., 1995a; Yuan et al., 1995b; Yuan and Schoenwolf, 1998; Davidson et al., 1999; Healy et al., 2001; Joubin and Stern, 1999; Joubin and Stern, 2001). Strikingly, the organizer can still form even when the entire territory containing cells that normally give rise to the organizer is removed (Joubin and Stern, 1999), including those derived from the sickle at very early stages (the early *GSC*-expressing population) and those recruited later (see above). Tissue recombination experiments showed that signals that define the organizer arise from surrounding tissues, notably a portion of primitive streak lying just caudal to the tip which emits “inducing” signals including cVg1/GDF3 and more peripheral regions outside the primitive streak that emit inhibitory signals including BMPs; canonical Wnt signalling also appears to play a role (Yuan et al., 1995b; Yuan and Schoenwolf, 1998; Joubin and Stern, 1999). Initially these observations suggested a remarkable ability of the organizer to regenerate. However, together with other findings discussed in this review, it became clear that what appeared to be regeneration is a manifestation of the normal process of cell recruitment into the organizer by cell movements. Therefore, the organizer emerges as a property of cells occupying a particular region of the embryo at a particular time (when they can receive appropriate instructive signals) that cause them to acquire characteristic patterns of gene expression and cell behaviour and to give rise to specific tissue type derivatives, rather than a special population of cells that is set aside early in development by particular combinations of transcription factors. Thus, the concept emerges that “*gene expression defines cell states, rather than cell fates*” (Solovieva et al., 2022a; Solovieva et al., 2022b; Joubin and Stern, 1999; Joubin and Stern, 2001), consistent with the dynamic features of gene expression in the organizer presented above, the dual (or more) sources of cells that generate the organizer, and the ability of the organizer to define a population of resident stem cells. This conclusion is worth taking into consideration when interpreting the results of single cell RNA sequencing in terms of either the origin, or the potential, or the subsequent fate of different cell types based exclusively on the expression of specific combinations of transcription factors at a particular instant in time (Stern, 2022; Stern, 2019).

## Cell heterogeneity in the organizer in relation to its functions

The studies discussed above already suggest that the organizer comprises several different subpopulations of cells, with different origins, different behaviours, and different fates. They also suggest that the tip of the streak/node is subdivided into at least a few domains where some of these properties are concentrated. One study subdivided the tip of the primitive streak at stage HH3^+^/4^−^ into 4 pieces (epiblast or deep, median/anterior or posterior-lateral) and investigated their ability to induce a patterned neural plate upon transplantation to a competent region of epiblast in a host embryo (Storey et al., 1995). Only the epiblast of both quadrants and the deep portion of the median quadrant can induce neural tissue, and only explants containing epiblast can both induce and pattern the host ectoderm (Storey et al., 1995). The ability of node cells to induce and pattern was also associated with the tissues to which the node sectors contributed: precursors of prechordal and axial mesoderm and of the endoderm were strongly associated with these classical organizer functions, whereas somitic mesoderm was not.

More recent studies have revealed that the tip of the streak/node is even more heterogeneous and suggested that some of this heterogeneity appears to be stochastic, with cells expressing different markers distributed in a “salt-and-pepper” pattern within the node (Solovieva et al., 2022a; Wood et al., 2024; Lu et al., 2025). The heterogeneity extends to the expression of signalling molecules, and correlative analyses of single cell mRNA expression shows that at least some of this heterogeneity is associated with specific transcription factor signatures (Lu et al., 2025). On the other hand, several of the original “markers” of the organizer, such as *GSC* (see above) are clearly expressed in all cells, confirming that the structure we usually identify as the organizer (both spatially and temporally) does indeed have an underlying molecular foundation, even if this underlies a more complex population structure. This latter study also suggested that while many of the targets of the organizer in neural induction (Trevers et al., 2023; Trevers et al., 2018) are regulated by signalling molecules that are expressed broadly in the organizer, such as *FGF8* and BMP inhibitors, some components of the neural induction network (Trevers et al., 2023) are regulated only by components expressed heterogeneously in the anterior primitive streak. These findings suggested the idea that the tip of the primitive streak might best be viewed as a “cell collective”, comprising cells with different specialised functions, which together account better for the diversity of functions associated with the organizer. This is also consistent with the finding discussed above that neither the “posterior population” nor the “central population” of organizer precursors alone have neural inducing ability, but do so when grafted together in a neural induction assay (Streit et al., 2000). This is an interesting area that should be the focus of future investigations, both in the chick and in other vertebrates.

## Other cell movements within and around the organizer: Left-right patterning

In addition to its neural induction and neural and mesodermal patterning functions along the rostrocaudal (neural plate) and mediolateral axes (mesoderm), the node is also associated with imparting left-right information to neighbouring tissues. Interestingly, there are significant differences in this among different vertebrate Classes, both relating to whether or not the organizer plays a role in left-right asymmetry at all, and to the mechanisms involved in those species where it does. In the chick, the node does not appear to be ciliated (a mechanism involved in asymmetry generation in mouse and human embryos), but it is molecularly asymmetric. The most important components of this signalling have been shown to include the “activin” (actually Nodal) receptor *ACVR2A* (on the right of the primitive streak, from stage HH 3^+^/4), Sonic hedgehog (*SHH*) and Cerberus-like (*CER1*), both on the left side of the node starting at HH 4/4^+^ (Zhu et al., 1999b; Rodriguez Esteban et al., 1999; Yokouchi et al., 1999; Levin et al., 1995; Stern et al., 1995), and *NODAL* itself in epiblast cells adjacent to the node on the left (from HH4^+^) then later (HH 5–6) in the left lateral mesodermal wing. As the role of signalling in left-right asymmetry in chick embryos has been reviewed extensively (Levin, 2005; Raya and Izpisua Belmonte, 2004), we will concentrate the remaining part of this section on the cellular aspects of this process in relation to the role of the node.

Observations of chick embryos from the 19th Century by August Kölliker (Kölliker, 1879) and Oscar Hertwig (Hertwig, 1898), and in the early years of the following century by Wetzel (Wetzel, 1929) already noted a marked morphological asymmetry in the node itself, whereby at head process stages (HH5–6) it appears to be tilted towards the left, with the emerging head process/notochord emerging primarily from that side (Fig. 4). In fact, the finding that *SHH* is expressed on the left side of the node by Mike Levin and Cliff Tabin (Levin et al., 1995) seemed as if it could be related to this morphological asymmetry, and it was the almost simultaneous finding that the receptor *ACVR2A* is expressed on the right of the streak and node (Levin et al., 1995; Stern et al., 1995) that suggested that the molecular asymmetry could not be ascribed exclusively to morphology, leading to a functional investigation of the relationships between these signals (Levin et al., 1995). Indeed, a few years later, when many more molecular asymmetries had been found, it was reported that morphological asymmetry precedes molecular asymmetries in developmental time (Männer, 2001; Dathe et al., 2002). A key finding (Gros et al., 2009) was that cells in the node and the surrounding epiblast undergo anti-clockwise rotation at the full primitive streak stage to generate the morphological asymmetry, and it was suggested that the movements are also the cause of the molecular asymmetries observed (Gros et al., 2009; Tsikolia et al., 2012) (Fig. 4). However, some early asymmetries, including the right-sided expression of *ACVR2A*, appear to arise not in the node but in the primitive streak, which does not experience these rotation movements, and the asymmetry is first visible at stage 3^+^/4^−,^ before the movements begin (stage 4^+^) (Stern et al., 1995). It was also suggested that at least some of the asymmetries in the node may originate in other neighbouring tissues, especially the presomitic mesoderm, and only later transferred to the node (Pagan-Westphal and Tabin, 1998). Thus, although a large-scale pattern of movements clearly does generate morphological asymmetry and at least maintains or enhances molecular asymmetries, there seems to be a complex interplay between signalling and morphogenesis that contributes to the patterning of the left-right axis and laterality of the body plan. What is even more puzzling however is how much this process seems to differ among different vertebrates. In some, like teleost fish and probably at least some amphibians, the “primary” or Spemann organizer does not appear to be involved at all. This raises interesting evolutionary questions, such as: how could a conserved pattern, like left-right asymmetries, be generated in evolution by radically different mechanisms in closely related vertebrate Classes? How many changes need to take place at the same time for one mechanism to be transformed into another?

## Conclusions

In this brief review I have tried to discuss some aspects of the biology of the organizer that involve cells and their behaviours, rather than only signalling molecules, which had until now been the strongest focus of investigations on the organizer in most species. It is becoming clear that the anterior primitive streak and node are regions of intense cellular activity and that these behaviours evolve as the functions of the organizer change over developmental time. In future it will be particularly interesting to investigate the relationships between signalling and different aspects of these cell behaviours, to establish the relative roles of each in the inducing and patterning functions of the organizer.
